# Seroprevalence of hepatitis E virus in dromedary camels, Bedouins, Muslim Arabs and Jews in Israel, 2009–2017

**DOI:** 10.1017/S0950268819000062

**Published:** 2019-02-22

**Authors:** R. Bassal, M. Wax, R. Shirazi, T. Shohat, D. Cohen, D. David, S. Abu-Mouch, Y. Abu-Ghanem, E. Mendelson, Z. Ben-Ari, O. Mor

**Affiliations:** 1 Israel Center for Disease Control, Ministry of Health, The Chaim Sheba Medical Center, Tel Hashomer, Ramat-Gan, Israel; 2 Central Virology Laboratory, Ministry of Health, Public Health Services, The Chaim Sheba Medical Center, Tel Hashomer, Ramat-Gan, Israel; 3 Sackler School of Medicine, Tel-Aviv University, Tel-Aviv, Israel; 4 Department of Virology, Kimron Veterinary Institute, Beit Dagan, Israel; 5 Liver Unit, Hillel Yaffe Medical Center, Hadera, Israel; 6 Department of Urology, The Chaim Sheba Medical Center, Tel Hashomer, Ramat-Gan, Israel; 7 Liver Disease Center, The Chaim Sheba Medical Center, Tel Hashomer, Ramat-Gan, Israel

**Keywords:** Arabs (Muslims, non-Bedouins), Bedouins, dromedary camels, hepatitis E, jews, seroprevalence, zoonozis

## Abstract

Hepatitis E virus (HEV) is an emerging cause of viral hepatitis worldwide. Recently, HEV-7 has been shown to infect camels and humans. We studied HEV seroprevalence in dromedary camels and among Bedouins, Arabs (Muslims, none-Bedouins) and Jews and assessed factors associated with anti-HEV seropositivity. Serum samples from dromedary camels (*n* = 86) were used to determine camel anti-HEV IgG and HEV RNA positivity. Human samples collected between 2009 and 2016 from >20 years old Bedouins (*n* = 305), non-Bedouin Arabs (*n* = 320) and Jews (*n* = 195), were randomly selected using an age-stratified sampling design. Human HEV IgG levels were determined using Wantai IgG ELISA assay. Of the samples obtained from camels, 68.6% were anti-HEV positive. Among the human populations, Bedouins and non-Bedouin Arabs had a significantly higher prevalence of HEV antibodies (21.6% and 15.0%, respectively) compared with the Jewish population (3.1%). Seropositivity increased significantly with age in all human populations, reaching 47.6% and 34.8% among ⩾40 years old, in Bedouins and non-Bedouin Arabs, respectively. The high seropositivity in camels and in ⩾40 years old Bedouins and non-Bedouin Arabs suggests that HEV is endemic in Israel. The low HEV seroprevalence in Jews could be attributed to higher socio-economic status.

## Introduction

Hepatitis E virus (HEV) is an emerging pathogen and one of the causes of viral hepatitis in the world [1]. The infection is mostly silent, but when symptoms do appear, illness is usually self-limiting [1]. HEV infection can also become chronic in immunocompromised individuals, such as those receiving organ transplants or chemotherapy, as well as among individuals with HIV infection [2]. Moreover, extra hepatic manifestations of HEV infection, mostly neurological, are increasingly recognised [3]. Currently, eight HEV genotypes are known, all belonging to a single serotype [4]. Of those, HEV-7 and HEV-8 have been identified in dromedary (1-humped) and bactrian (2-humped) camels, respectively [4, 5]. HEV-7, identified in a liver transplant recipient had been linked to the consumption of camel products [6].

Human HEV infection, investigated using seroprevalence studies, was found to be more prevalent in older ages [7–20], lower socio-economic status [21], poorer residence areas [9, 14–15], among sheltered homeless adults [22] or uneducated people [14], specific nationalities (for example, higher in mixed race donors and ethnic groups within China [12, 15], or in immigrants from Afghanistan [14]), drinking water from wells or rivers [15], consumption of meat products [7, 15, 17, 23] especially pork [24, 25] and following blood transfusions [1].

The majority of the population in Israel comprises Jews (74.8%) and Arabs (20.8%), of which 84.7% are Muslims [26]. While almost all Israeli-Arabs were born in Israel, Jews compose a mixed population of Israeli-born and non-Israeli-born individuals. Previously, we have shown that the overall HEV seroprevalence in Israel was 10.6% and higher in Arabs (22.5%) compared with Jews (10.3%); with most of the anti-HEV positive Jews being immigrants not born in Israel [21]. However, an assessment of anti-HEV prevalence in specific subgroups of Arabs has not been done.

The Bedouins in Israel compose a unique nomadic Muslim Arab population with cultural, historical and social uniqueness. While non-Bedouin Muslim Arabs live in villages spread around the country, mostly in the northern and central part of Israel and are not physically related to camels, many of the Bedouins who live in southern Israel own dromedary camels and consume camel products [27].

This study aimed to assess HEV seroprevalence in camels (dromedary), in Bedouins living in the southern part of Israel in the vicinity of camels, in non-Bedouins Arabs (Muslims living in northern and central Israel) and in Israeli-born Jews and to assess the factors associated with anti-HEV seropositivity.

## Methods

*Camel serum samples* (*n* = 86) from female dromedary camels aged 3 (*n* = 43), 7 (*n* = 25) and 10 and above years (*n* = 18) were collected between 2015 and 2017 from Bedouin households residing in the southern district of Israel (as part of the Middle East Respiratory Syndrome (MERS) National Control Programme) and were used for determining HEV seroprevalence in the local 1– humped camels.

*Human serum samples* were collected from: Bedouins living in the southern district, in tribes that own dromedary camels (overall 305; 296 tested for the first time in this study and nine added from our previous study [21]), non-Bedouin Arabs (overall 320; 297 from the present study and 23 from our previous study [21]) and from Jews (195 samples presented in our previous study [21]). All human samples were randomly selected using an age-stratified sampling design from the stored sera bank of the Israeli Centre for Disease Control. The samples were selected from those collected between 2009 and 2016, >20 years old who were born in Israel. The selection was enabled by the availability of basic demographic information including age, gender, place of residence (city), birth country and population group (Bedouins; non-Bedouin Arabs; Jews [28]) for each sample in this sera bank. The socio-economic status was allocated on the basis of the given address using the socio-economic residential classification published by the Israeli Central Bureau of Statistic [29]. This socio-economic status is based on 14 variables including demographic characteristics, education, lifestyle, etc. and was divided into low (1–5) *vs.* high (6–10).

Sera collection was approved by the Legal Department of the Israeli Ministry of Health.

### Laboratory analysis

Total anti-HEV antibodies were assessed in camel samples using HEV-Ab ELISA kit (Wantai, Biologic Pharmacy Enterprise, Beijing, Republic of China) which detects total antibodies and is suitable for detection of anti HEV antibodies in non-human sera. HEV RNA in camel sera was assessed with the RealStar HEV kit (Altona Diagnostics GmbH, Hamburg, Germany) which, according to the manufacturer, should detect all HEV genotypes. Human samples from the current study were tested with anti-IgG HEV ELISA kit (Wantai, Biologic Pharmacy Enterprise, Beijing, Republic of China) which recognises human antibodies against all HEV genotypes with 97.96% sensitivity and 99.99% specificity [30]. Assays were performed according to the manufacturer's instructions. All serological equivocal results were considered as negative. In the previous study, we used the DSI-Anti-HEV-IgG kit (Diagnostic Systems Italy, Saronno, Italy) [21]. Merging the current and previous study data were applicable after comparing the kits performances using 90 positive and negative samples, revealing 95.6% concordance between the kits. HEV RNA was not assessed in human sera due to lack of sufficient serum material.

### Data analysis

Descriptive analysis was done for the study populations. Prevalence rates of anti-HEV antibodies in camels and in human sera were calculated by dividing the number of samples positive to anti-HEV antibodies by the total number of samples tested in each group. For the human samples, we calculated the prevalence rates in each of the studied populations by age group, gender and socio-economic status. We used the Cochran-Armitage Trend Test to evaluate trends in binomial proportions and the *χ*^2^ test to compare between population groups. Logistic regression analyses were applied to assess the factors associated with anti-HEV seropositivity. Interaction was assessed for each variable associated with HEV seropositivity. Statistical significance was evaluated using 2-sided tests with an alpha level of 0.05. All analyses were performed using SAS Enterprise Guide (version 7.12 HF5, SAS Institute Inc., Cary, NC, USA).

## Results

### Camels’ samples

Of the samples obtained from camels, 68.6% (95% CI 57.7–78.2%) were anti-HEV IgG positive. The seroprevalence among camels 10 and above, 7 and 3 years old were 88.9% (16/18) (95%CI 65.3–98.6%), 56.0% (14/25) (95% CI 34.9–75.6%) and 67.4% (29/43) (95% CI 51.5–80.9%). None of the samples were HEV RNA positive.

### Humans’ samples

Table 1 demonstrates the demographic characteristics and the seropositivity rates of each population group. The age range of Bedouins was 20.0–79.6 years, non-Bedouin Arabs: 20.1–88.1 years and Jews: 20.0–96.8 years. Overall, 21.6% of the samples obtained from Bedouins (95% CI 17.2–26.7%), 15.0% of the samples obtained from non-Bedouin Arabs (95% CI 11.3–19.4%) and 3.1% of the samples obtained from Jews (95% CI 1.1–6.6%) were anti-HEV IgG positive. HEV seropositivity was significantly higher in Bedouins and in non-Bedouin Arabs compared to Jews (*P*-value < 0.001). The difference in seropositivity rates between Bedouins and non-Bedouin Arabs was statistically significant (*P*-value = 0.032).

**Table 1.** Demographic characteristics distribution and seroprevalence of human serum samples in Israel of anti-Hepatitis E IgG positive, by population group

			Tested		Positive	
Population Group	Variable	Category	*N*	%	*n*	% (95% CI)
Bedouins	Age group (years)	20–29	101	33.1	1	1.0 (0.0–5.4)
		30–39	101	33.1	16	15.8 (9.3–24.5)
		⩾40	103	33.8	49	47.6 (37.6–57.7)
	Gender	Male	155	50.8	35	22.6 (16.3–30.0)
		Female	150	49.2	31	20.7 (14.5–28.0)
	Socio-economic status	1–5	143	46.9	37	25.9 (18.9–33.9)
		6–10	0	0.0	0	0.0
		Missing	162	53.1	29	17.9 (12.3–24.7)
Non-Bedouin Arabs	Age group (years)	20–29	104	32.5	1	1.0 (0.0–5.2)
		30–39	104	32.5	8	7.7 (3.4–14.6)
		⩾40	112	35.0	39	34.8 (26.1–44.4)
	Gender	Male	160	50.0	21	13.1 (8.3–19.4)
		Female	160	50.0	27	16.9 (11.4–23.6)
	Socio-economic status	1–5	300	93.8	44	14.7 (10.9–19.2)
		6–10	9	2.8	2	22.2 (2.8–60.0)
		Missing	11	3.4	2	18.2 (2.3–51.8)
Jews	Age group (years)	20–29	40	20.5	1	2.5 (0.1–13.2)
		30–39	46	23.6	0	0.0 (0.0–7.7)
		⩾40	109	55.9	5	4.6 (1.5–10.4)
	Gender	Male	109	55.9	2	1.8 (0.2–6.5)
		Female	86	44.1	4	4.6 (1.3–11.5)
	Socio-economic status	1–5	50	25.6	2	4.0 (0.5–13.7)
		6–10	93	47.7	3	3.2 (0.7–9.1)
		Missing	52	26.7	1	1.9 (0.0–10.3)

Table 2 demonstrates the odds for being HEV positive, by population group, demonstrating significantly higher odds among the older age groups of both Arab populations.

**Table 2.** Single variable analysis of human serum samples in Israel of anti-Hepatitis E IgG positive, by population group

Population group	Variable	Category	OR	95% CI	*P*-value
Bedouins	Age group (years)	20–29	Ref.	–	–
		30–39	18.82	2.44–144.89	0.005
		⩾40	90.74	12.19–675.42	<0.001
	Gender	Male	1.12	0.65–1.93	0.685
		Female	Ref.	–	–
Non-Bedouin Arabs	Age group (years)	20–29	Ref.	–	–
		30–39	8.58	1.05–69.79	0.044
		⩾40	54.98	7.39–408.93	<0.001
	Gender	Male	0.74	0.40–1.38	0.349
		Female	Ref.	–	–
	Socio-economic status	1–5	0.60	0.12–2.99	0.534
		6–10	Ref.	–	–
Jews	Age group (years)	20–29	Ref.	–	–
		30–39	<0.01	<0.01->999.99	0.958
		⩾40	1.88	0.21–16.56	0.572
	Gender	Male	0.38	0.07–2.14	0.275
		Female	Ref.	–	–
	Socio-economic status	1–5	1.25	0.20–7.74	0.810
		6–10	Ref.	–	–

A single variable analysis demonstrated that seropositivity was significantly higher among Arabs (Bedouins and non-Bedouin); older age groups (30–39 and ⩾40 years) and lower socio-economic status (Table 3). No significant interaction was identified. These associations remained statistically significant in the multivariable analysis, except for the lower socio-economic status (Table 3).

**Table 3.** Single variable and multivariable analysis of factors associated with anti-HEV seropositivity

		Single variable analysis^a^			Multivariable analysis^b^		
Variable		OR	95% CI	*P*-value	OR	95% CI	*P*-value
Population group	Jews	Ref.	–	–			
	Bedouins	8.70	3.69–20.50	<0.001	24.02	6.62–87.18	<0.001
	Non-Bedouin Arabs	5.56	2.33–13.25	<0.001	7.75	2.32–25.88	0.001
Age group (years)	20–29	Ref.	–	–			
	30–39	8.53	2.53–28.70	0.001	10.82	2.43–48.26	0.002
	⩾40	32.47	10.14–103.96	<0.001	77.57	18.23–330.07	<0.001
Gender	Female	Ref.	–	–			
	Male	0.85	0.58–1.26	0.424	0.80	0.47–1.36	0.400
Socio-economic status	1–5	3.93	1.55–9.45	0.004	1.24	0.35–4.38	0.736
	6–10	Ref.	–	–			

a In the single variable analysis, 820 samples were included for population group, age group and gender analyses and 595 in the socio-economic status.

b In the multivariable analysis, 595 samples were included.

In a sensitivity analysis performed for the classification of the equivocal samples as positive or negative, no significant difference was observed.

## Discussion

HEV prevalence among dromedary camels, has been studied in the past and following literature review we realise that our finding (68.6%) is higher than the 22.4% seroprevalence rates reported in Ethiopia [31], but similar to the 62.9% seropositivity among dromedary camels recently reported in Egypt, a nearby country [32]. Together, these results suggest that the 1-humped camels in our region are highly seropositive for HEV.

Both Bedouins (21.6%) and non-Bedouin Arabs (15.0%) were characterised by significantly higher HEV seroprevalence rates compared with the overall low seropositivity rates (3.1%) observed in Jews. Together with the high seroprevalence of HEV in camels, these results indicate endemicity of HEV in Israel. Worldwide, the seroprevalence of HEV documented in human populations varies between high (⩾20.0%), medium (10.0–19.9%) and low (<10.0%). High HEV seroprevalence rates were reported in Uganda (47.7%) [33], Poland (43.5% and 49.6%) [9, 18], Bolivia (34.8%) [13], Jordan (30.9%) [7] and South Africa (25.3%) [12]. Medium HEV seroprevalence rates were reported in China (19.9%) [15], Belgium (18.3%) [34], Portugal (16.3%) [11] and the Mediterranean Region (11.9%) [35]. Low HEV seroprevalence rates were reported in New Zealand (9.7%) [10], Scotland (6.1%) [36] and Iceland (2.1%) [37]. Accordingly, within Israel, high, medium and low HEV seroprevalence rates were observed in Bedouins, non-Bedouin Arabs and Jews.

The higher seroprevalence rate in Bedouins could be attributed to their low socio-economic status but could also result from exposure to camels, especially as the latter were highly seroprevalent (possibly a consequence of HEV-7 infection [4]). As camel samples were HEV RNA negative and fecal specimens were not available for analysis, this hypothesis could not be further explored. In Jordan, a nearby country, an overall of 30.9% anti-HEV prevalence rate was determined in the local population and owning camels was associated with increased odds of HEV seropositivity [7]. Future studies should aim to assess the HEV RNA prevalence in camel's feces and in their fresh blood samples, as well as in similar samples obtained from Bedouins who own camels. With the recent report suggesting that HEV originated in Asia, most likely from a human ancestor that existed ~4500–6800 years ago and that the split of camel-infecting genotypes occurred during camel domestication, HEV circulation between camels and Bedouins could be expected [38]. The high HEV seropositivity rate observed in non-Bedouin Arabs, most of whom live in Northern parts of Israel, could be attributed to exposure to HEV-3 which is possibly circulating in the country and was recently identified in local sewage facilities mainly in the north of Israel [39]. As both Judaism and Islam forbid the consumption of pork, we find it unlikely that this is the main risk for the identified HEV seroprevalence in the Muslim population. The low HEV seroprevalence observed in Israeli-born Jews may be associated with the overall higher socio-economic status characterising this population, living in better sanitary conditions than the Bedouins and most of the non-Bedouin Arabs, rendering HEV infection less conceivable.

The variability observed between different population groups and in different studies, may be explained by exposure to potential risk factors associated with HEV infection as lifestyle habits (dietary), environmental conditions, geographic location, occupation, religion (as the prohibition of consuming pork among Jews and Islam) but also to co-morbidities. Differences may also be explained by the assays used for antibodies detection, demonstrating wide variation in the ability to detect HEV antibodies (sensitivity and specificity) [40].

The association of HEV seropositivity with age observed in our study has also been reported by others [7–20] and may be explained not only by the cumulative lifetime exposure to HEV, but also by cohort effect, where certain population groups were more likely to be exposed to the virus during their life.

The major limitation of our study is the lack of data on characteristics that might be important to exposure to HEV, such as actual exposure to animals, consumption of camel meat and the quality of the local water source. Additionally, the cross-sectional nature of the data cannot establish a temporal relationship between risk factors and outcome, thus limits the interpretation of the results. Finally, HEV RNA from human samples could not be assessed due to limited serum volumes. However, for the first time, we have investigated samples from local camels and from specific human populations, with fair distribution by age groups and gender. Based on this cohort, our results suggest that HEV is endemic in Israel and that specific population groups like Bedouins and non-Bedouin Arabs are at higher risk of HEV infection. Further studies are needed to determine the HEV genotypes circulating among dromedary camels and the specific Arabs populations.
